# Effects of gibberellins on important agronomic traits of horticultural plants

**DOI:** 10.3389/fpls.2022.978223

**Published:** 2022-10-04

**Authors:** Xiaojia Zhang, Baolin Zhao, Yibo Sun, Yulong Feng

**Affiliations:** ^1^ Liaoning Key Laboratory for Biological Invasions and Global Changes, College of Bioscience and Biotechnology, Shenyang Agricultural University, Shenyang, China; ^2^ Chinese Academy of Science (CAS) Key Laboratory of Tropical Plant Resources and Sustainable Use, CAS Center for Excellence in Molecular Plant Sciences, Xishuangbanna Tropical Botanical Garden, Chinese Academy of Science, Kunming, China

**Keywords:** horticultural plants, gibberellins, plant stature, flowering time, parthenocarpy

## Abstract

Horticultural plants such as vegetables, fruits, and ornamental plants are crucial to human life and socioeconomic development. Gibberellins (GAs), a class of diterpenoid compounds, control numerous developmental processes of plants. The roles of GAs in regulating growth and development of horticultural plants, and in regulating significant progress have been clarified. These findings have significant implications for promoting the quality and quantity of the products of horticultural plants. Here we review recent progress in determining the roles of GAs (including biosynthesis and signaling) in regulating plant stature, axillary meristem outgrowth, compound leaf development, flowering time, and parthenocarpy. These findings will provide a solid foundation for further improving the quality and quantity of horticultural plants products.

## Introduction

Gibberellins (GAs) are numerous plant hormones that are all consist of a large number of diterpenoid compounds (Hedden, 2020; Zhang et al., 2020). GA was first characterized from the pathogenic fungus *Gibberella fujikuroi* by Japanese scientists (Yabuta, 1938; Binenbaum et al., 2018), and it causes severe disease with the symptom of excessive internode elongation known as ‘foolish seedling disease’ in *Oryza sativa* (rice). More than 130 GAs have been discovered in plants, fungi, and bacteria (Yamaguchi, 2008; Hedden, 2020), and they are named GAn (for example GA_4_) in the order of discovery (MacMillan and Takahashi, 1968). Among GAs, only GA_1_, GA_3_, GA_4_, and GA_7_ are bioactive, controlling multiple developmental processes in plants (Yamaguchi, 2008; Hedden, 2020; Zhang et al., 2020).

**Figure 1:**
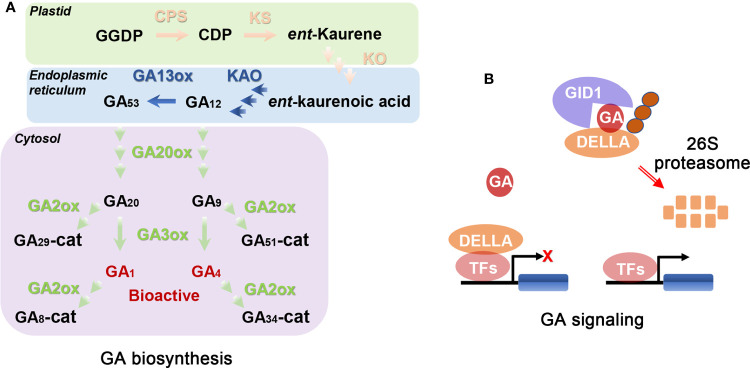
Schematic representations of GA biosynthesis **(A)** and signal **(B)** pathways (Yamaguchi, 2008; Binenbaum et al., 2018; Wu et al., 2020). **(A)** GGDP, CDP, and cat represent geranylgeranyl diphosphate, *ent*-copalyl diphosphate, and catabolite, respectively. CPS, KS, KO, and KAO represent *ent*-copalyl diphosphate synthase, *ent*-kaurene synthase, *ent*-kaurene oxidase, and *ent*-kaurenoic acid oxidase, respectively. GA13ox, GA20ox, GA3ox, and GA2ox represent GA13-oxidases, GA20-oxidases, GA3-oxidases, and GA2-oxidases, respectively. CPS, KS, KO, KAO, GA13ox, GA20ox, and GA3ox are the key enzymes in GA biosynthetic pathway. GA2ox is GA deactivated enzyme. Enzymes locate in plastid, endoplasmic reticulum, and cytosol using pink font, green font, and blue font, respectively. Bioactive metabolic products (GA_1_ and GA_4_) use red font. The remaining metabolic products use black font. **(B)** When bioactive GAs are exhausted or limited, DELLAs (consisting of the following proteins: GA-INSENSITIVE, GAI; REPRESSOR OF GA1-3, RGA; RGA-LIKE1, RGL1; RGL2 and RGL3; GRAS-domain proteins) interact with transcription factors (TFs) inhibiting expression of corresponding downstream genes and therefore growth. When endogenous GA concentration is increased, a complex of GA-INSENSITIVE DWARF1 (GID1; the GA receptor)-GA-DELLA is formed. Then, the complex is recognized by the F-box proteins, leading to degradation of DELLA by ubiquitin-26S proteasome system and thus releasing the growth inhibition. GID1 and DELLAs are the core components of GA signal pathway. The orange circles represent F-box proteins, such as SLEEPY1 (SLY1).

It is important to study the biosynthesis and signaling pathways of GAs to determine their biological functions. GA biosynthesis in plants consists of a series of complicated oxidation-reduction reactions, including reactions from geranylgeranyl diphosphate (GGDP) to bioactive GAs (Yamaguchi, 2008; Hedden, 2020). This process is usually classified into three stages in *Arabidopsis* (**Figure 1A**; Yamaguchi, 2008; Zhang et al., 2020; Cheng et al., 2021; Shohat et al., 2021). The first stage is the synthesis of GGDP to *ent*-kaurene using GGDP as starting material in plastids, which is consecutively catalyzed and oxidated by *ent*-copalyl diphosphate synthase (CPS) and *ent*-kaurene synthase (KS) (**Figure 1A**; Sun and Kamiya, 1997; Helliwel et al., 2001; Hedden, 2020). The second stage is the conversion from *ent*-kaurene to GA_12_ in six steps. The first three steps are oxidized by *ent*-kaurene oxidase (KO) and the remaining steps by *ent*-kaurenic acid oxidase (KAO) (**Figure 1A**; Regnault et al., 2014). KO is located in the plastid membrane while KAO is in the endoplasmic reticulum (Yamaguchi, 2008). GA biosynthesis from GA_12_ to bioactive GAs is the last stage, which is divided into the 13-hydroxylated pathway (from GA_53_ to GA_1_) and the non-13-hydroxylated cascade (from GA_12_ to GA_4_). In the beginning, partial GA_12_ is converted to GA_53_ catalyzed by GA 13-oxidases (GA13ox) (Magome et al., 2013; He et al., 2019a). Then, GA_12_ and GA_53_ are converted to bioactive GAs in the cytosol after a series of oxygenation reactions catalyzed mainly by GA 20-oxidases (GA20ox) and GA 3-oxidases (GA3ox; **Figure 1A**; Tenreira et al., 2017; Zhang et al., 2020).

Among the genes encoding the above-mentioned GA biosynthetic enzymes, *CPS*, *KS*, and *KO* are single copy genes, while the others are multi-copy genes. Among the above-mentioned enzymes of GA biosynthesis, GA20ox is a rate-limiting enzyme (Yamaguchi, 2008). Mutations of GA biosynthesis related genes decrease plant concentrations of bioactive GAs, leading to multiple defective phenotypes (Zhang et al., 2020). These defective phenotypes can be recovered by addition of exogenous bioactive GAs, and hence these mutants are called GA-deficient mutants. Besides GA biosynthesis, GA deactivation is also important for modifying the concentrations of bioactive GAs in plant tissues (Schomburg et al., 2003; Zhu et al., 2006). For example, GA 2-oxidases (GA2ox) can catalyze GA_20_, GA_9_, GA_1_, and GA_4_ to inactive GAs (**Figure 1A**). Loss of function of GA deactivated genes often leads to tissues with high concentrations of bioactive GAs.

The GA signaling pathway has also been characterized in *Arabidopsis*. The current model of the GA signaling pathway consists of several key components, including bioactive GAs, GIBBERELLIN INSENSITIVE DWARF1 (GID1, the GA receptor), DELLAs (consisting of the following proteins: GA-INSENSITIVE, GAI; REPRESSOR OF GA1-3, RGA; RGA-LIKE1, RGL1; RGL2 and RGL3; GRAS-domain proteins), and two F-box proteins (SLEEPY1; SLY1 and SNEEZY; SNZ) (Davière and Achard, 2014; Shohat et al., 2021). When bioactive GAs are exhausted or limited, DELLAs interact with multiple transcription factors (TFs), inhibiting expression of corresponding downstream genes, and thus repressing almost all GA-related growth responses (Davière and Achard, 2014; Shohat et al., 2021; Chai et al., 2022; Jin et al., 2022). When bioactive GA concentration is increased, a GA-binding pocket in GID1 captures bioactive GAs and H_2_O, causing conformational change of the flexible N-terminal extension from the pocket and thus closing the GA-binding pocket (Murase et al., 2008; Shimada et al., 2008). Subsequently, DELLAs bind with the N-terminal extension of GID1 *via* its TVHYNP regions forming a GID1-GA-DELLA complex, and the recruited F-box proteins immediately bind with DELLA (Shohat et al., 2021). SLY1 (a F-box protein) is a component of SKP1, CULLIN, F-BOX (SCF) E3 ubiquitin-ligase complexes and the SCF complex contributes to subsequent degradation of DELLAs. Once the GID1-GA-DELLA-SCF^SLY1^ complex is formed, DELLAs are subsequently degraded by the 26S proteasome, releasing the growth inhibition (**Figure 1B**; Davière and Achard, 2014; Shohat et al., 2021; Chai et al., 2022). DELLAs exert a core role in the GA signaling pathway. Mutations of GA perception and signal transduction can lead to mutant phenotypes in plants even if they have high concentrations of GAs. This kind of defective phenotype of mutant plants cannot be recovered using exogenous bioactive GAs, and they are hence called GA-insensitive mutants.

Both GA-deficient and GA-insensitive mutants generally exhibit typical dwarf phenotypes (Davière and Achard, 2014; Hedden, 2020; Zhang et al., 2020). Among these mutants, mutations of *CPS*, *KS*, or *KO* always lead to severe dwarfism because they are all single-copy genes (Sun and Kamiya, 1997; Helliwel et al., 2001; Regnault et al., 2014). Mutations of other GA-biosynthesis and -signaling pathway related genes often display a semi-dwarf phenotype as they are all multi-copy genes (Griffiths et al., 2006; Magome et al., 2013). In comparison, mutants related to constitutively active GA exhibit a spindly internode, such as *elongated uppermost internode* (*eui*; a deactivated gene; Zhu et al., 2006). The *semi-dwarf1* (*sd1*) and *Reduced height-1*(*Rht-1*) mutants, caused by mutations of *GA20ox2* and *DELLA*, respectively, prevent excessive internode growth and lodging, and therefore grain yield loss caused by wind and overuse of chemical fertilizer (Peng et al., 1999; Sasaki et al., 2002). The dramatically increased yield from semi-dwarf crops has saved countless lives, and is referred to as the ‘Green Revolution’. Recently, the GROWTH-REGULATING FACTOR4 (GRF4)-DELLA-NITROGEN-MEDIATED TILLER GROWTH RESPONSE 5 (NGR5) module in rice has shown potential to improve nitrogen use-efficiency (NUE) and increase tiller number in semi-dwarf ‘Green Revolution’ varieties (Li et al., 2018a; Wu et al., 2020). This discovery is regarded as a new Green Revolution in the 21^st^ century.

Apart from biosynthetic and signaling pathways of GAs, the local accumulation maxima of bioactive GAs in the organ formation zone is a prerequisite for normal organ development (Binenbaum et al., 2018). Synthesis sites of bioactive GAs are generally the sites where GAs function. However, this is not always the case (Ragni et al., 2011; Lacombe and Achard, 2016; Camut et al., 2019). Hence, transportation of GAs from synthesis sites to the sites where they function is essential (Katsumi et al., 1983). Some precursors of bioactive GAs or their deactivated metabolites can also be transported (Hu et al., 2008; Regnault et al., 2015; Lange and Lange, 2016; Camut et al, 2019). Like other hormones, GAs can possibly move in both directions in vascular tissues (Binenbaum et al., 2018; Zhang et al., 2020). The transportation of GAs can be classified into short- and long-distance movements. The short-distance movement of GAs has been demonstrated in *Cucumis sativus* (cucumber; Hu et al., 2008; Lange and Lange, 2016). GA_9_ (a precursor of GA_4_; **Figure 1A**) is synthesized in cucumber ovaries where *GA20ox* has relatively high expression, while GA_9_ is converted to GA_4_ in sepals and petals where a relatively high expression level of *GA3ox* is detected (Lange and Lange, 2016). Thus, the short-distance movement of GA_9_ is essential for flower development in cucumber.

Many studies in *Arabidopsis* have demonstrated that the long-range transportation of endogenous GA_12_ enhances the ability to adapt to adverse environments (Regnault et al., 2015; Camut et al, 2019). The long-distance movement of GAs can even be root-to-shoot and shoot-to-root transportation (Regnault et al., 2015; Camut et al, 2019). It is generally believed that precursors of bioactive GAs are dominating forms of movement, although the mobility of bioactive GAs was identified approximately 40 years ago (Katsumi et al., 1983). A previous study showed that bioactive GA_3_ could be transported in grafts between normal and mutant seedlings in *Zea mays* (maize; Katsumi et al., 1983). Obviously, the movements of GAs are involved in controlling plant growth and development. But it is still not clear how GAs are transported from cell to cell and what their receptors are in cell membranes in horticultural plants and other species.

Horticultural plants include numerous species, such as *Solanum lycopersicum* (tomato), *Capsicum annuum* (peppers), *Pisum sativum* (pea), *Brassica rapa* L. ssp. *Pekinensis* (Chinese cabbage), *Lactuca sativa* (lettuce), *Malus pumila* (apple), *Vitis vinifera* (grape), *Rosa chinensis* (rose), and *Panax ginseng* (ginseng). The products of horticultural plants are not only served as foods, but also provide dietary intake of vitamins and minerals. Moreover, some secondary metabolites from horticultural plants are often used to treat human diseases. Horticultural plants are becoming more and more important for human beings under population increases, global environmental changes, and land degradation. Fortunately, genomic data of numerous horticultural species have been published in recent years (Sun et al., 2021), which is advantageous for horticultural researchers. However, studies on the roles of GAs in manipulating growth and development of horticultural plants are lacking compared with rice and *Arabidopsis*. In this review, we focus on GA biosynthesis and GA signaling in horticultural plants and discuss how GA regulates vegetative and reproductive growth of horticultural plants, with the purpose of helping horticultural researchers understand the genetic and molecular mechanisms of GA functions.

## Vegetative growth

### Plant stature

An ideal plant architecture is important in cereal crops but is also critical to horticultural plants. Shoot architecture is mainly influenced by stem elongation (Guo et al., 2020; Zhang et al., 2020). In the 1960s, the use of semi-dwarf cereal crop varieties contributed to great increases in crop yield (Peng et al., 1999; Sasaki et al., 2002). Subsequently, related mutation genes (*GA20ox2* and *DELLA*) were identified in rice and *Triticum aestivum* (wheat), and GAs were shown to be involved in the semi-dwarf trait (Peng et al., 1999; Sasaki et al., 2002). Similar results were also found in horticultural plants (Schrager-Lavelle et al., 2019; Sun et al., 2020).

By bulked segregant analysis (BSA) and mapping, the gene responsible for ‘w106 (dwarf)’ in *Citrullus lanatus* (watermelon) was identified as *Cla015407* encoding ClGA3ox (Sun et al., 2020). Similar results were found at the same time by Gebremeskel et al. (2019). The *tomato internode elongated-1* (*tie1*) mutants exhibit the internode elongation phenotype and this is a loss-of-function mutation of *SlGA2ox7* (a GA catabolic gene; Schrager-Lavelle et al., 2019). Paclobutrazol (PAC), a GA biosynthesis inhibitor, can restore the *tie1* defective phenotype (Schrager-Lavelle et al., 2019). The mutation of *Non-Heading Mutant* (*NHM1*; encoding KS enzyme) leads to the non-heading phenotype in Chinese cabbage (Gao et al., 2020). Reverse genetic screening results also verify that GAs positively control internode elongation. Editing *PROCERA*/*SlDELLA* in tomato and *MaGA20ox2* in *Musa acuminate* (banana) using CRISPR-Cas9 results in shortened internodes (a dominant mutation and a loss-of-function mutation; Tomlinson et al., 2019; Shao et al., 2020). Overexpression of *PsGA3ox1* and *PpGA2ox1* lead to longer and more compact internodes in pea and *Nicotiana tabacum* (tobacco), respectively (Reinecke et al., 2013; Cheng et al., 2021). GAs and PAC application also obviously change the internode length of two commercial grapevine cultivars (Acheampong et al., 2015).

All of the genes discussed above (*Cla015407*, *TIE1*, *NHM1*, *MaGA20ox2*, *PsGA3ox1*, and *PROCERA*) are classified into GA synthesis or signal genes directly influencing endogenous GA in tissues, and abnormal concentrations of endogenous GAs affect development of tissues, such as stem. We assume that this is also a conservative mechanism of horticultural species as in model plants where GAs positively regulate stem elongation (Tomlinson et al., 2019; Shao et al., 2020; Cheng et al., 2021; Kou et al., 2021; Liu et al., 2022). Internode/stem is made up of millions of cells, and cell fate determines internode elongation. Endogenous GAs influence cell fate (elongation or/and cell proliferation). Studies of shade avoidance syndrome provided solid evidence of how GAs precisely regulate cell fate (Roig-Villanova and Martínez-García, 2016; Lee et al., 2018). When endogenous GAs were elevated, *Xyloglucan endotransglucosylase*/*hydrolases* (*XTHs*), *Expansins* (*EXPs*), and *Cyclin-dependent kinases* (*CDKs*) were subsequently up-regulated. These genes acidize and soften cell walls, benefiting stem cell elongation and proliferation. In other words, internode elongation or shortening in horticultural plants are determined by cell development (elongation, division, and expansion), and cell fate is accurately regulated by fluctuations in endogenous GAs.

Although a few GA synthesis and metabolism genes have been verified to be involved in internode length, regulatory factors of GA synthesis and signal transduction are still poorly understood at present for horticultural plants. For example, MCMl AGAMOUS DEFICIENS SRF4 (MADS)-box and basic helix-loop-helix (bHLH) TFs, which have been frequently found in *Arabidopsis* and rice, are still lacking in horticultural plants. Identifying regulatory factors in GA biosynthesis and signaling pathways is a necessary future research topic in horticultural plants.

### Axillary bud outgrowth

Axillary bud outgrowth also significantly affects the quality and quantity of the products of horticultural plants, as it is another core factor determining plant architecture (Wang et al., 2018a; Zhang et al., 2020; Feng et al., 2021). The lateral axillary meristem in the axil of leaves of horticultural species first develops into axillary buds (namely, axillary bud initiation), and then the axillary bud develops into dormant buds, shoot branching/branch crown, or specialized tissues (runners etc.; Wang et al., 2018a; Zhang et al., 2020; Guo et al., 2021).

Clonal propagation (runners) produces offspring with the same genetic background (Tenreira et al., 2017) as their parents in *Fragaria vesca* (a diploid model plant of strawberry) or *Fragaria × ananassa* (a cultivated strawberry). It is important to analyze how the runners form. This process includes axillary bud initiation and outgrowth, which are both strictly controlled by internal factors such as GAs and TFs and external factors such as photoperiod (Mouhu et al., 2013; Tenreira et al., 2017; Caruana et al., 2018; Li et al., 2018b; Andrés et al., 2021; Feng et al., 2021; Guo et al., 2021; Liang et al., 2022). *LOSS OF AXILLARY MERISTEMS* (*LAM*, a GRAS gene) is highly expressed in axillary meristem. Mutation of *LAM* leads to decreased runner number due to a failure of axillary bud formation, indicating that *LAM* can provoke bud initiation in *F. vesca* (Feng et al., 2021). GA application only stimulates the remaining axillary bud outgrowth in *lam* plants but fails to recover the defect of axillary bud decrease, indicating that GAs exclusively induced runner development instead of bud initiation in *F. vesca* (Feng et al., 2021). A previous study demonstrated that *FveGA20ox4* is exclusively expressed in axillary meristem and the developmental runners (Tenreira et al., 2017). Mutation of *GA20ox4* leads to reduced runner number and the defective phenotype of the *r* mutant is rescued by exogenous GA_3_ in *F. vesca* (Tenreira et al., 2017). Apart from GA synthesis genes, GA signal transduction genes are also involved in axillary bud outgrowth. Loss of function or downregulation of *RGA1* (one of the five *DELLAs* in strawberry) increases runner number in *F. vesca*, which has been demonstrated by different research groups (Caruana et al., 2018; Li et al., 2018b).

In addition, TFs are also essential in runner formation. FaHANABA TARANU (FaHAN; a GATA TF) promotes runner development *via* enhancing GA concentration in *F. ananassa* (Liang et al., 2022). Surprisingly, the *GA20ox1* transcript is accumulated in *FaHAN*-OE transgenic lines, but the *GA20ox4* gene is not in *F. ananassa* (Liang et al., 2022). Long-day and 18°C are also beneficial to runner development in *F. vesca* (Mouhu et al., 2013; Andrés et al., 2021). Long-day positively regulates the FvFlowering Locus T (FT)–FvSUPPRESSOR OF OVEREXPRESSION OF CONSTANS1 (SOC1) cascade, and FvSOC1 directly up-regulates *FvGA20ox4* expression in *F. vesca*. These processes lead to increased bioactive GA concentration, and therefore promote runner development. Andrés et al. (2021) further found that there may be a FvSOC1-indpendent way in *F. vesca*, which controls runner development at temperature of 22°C.

In contrast, silencing the *SlGA2ox* gene inhibits shoot branching, and the role of GAs is verified by application of GAs and PAC in tomato, which proves that GAs play a negative role in shoot branching in tomato (Martínez-Bello et al., 2015). In garlic bulb, however, GAs promote axillary meristem initiation and determine the number of cloves (Liu et al., 2019; Liu et al., 2020). Application of GA_3_ results in downregulation of *AsGA20ox* and upregulation of *AsHistidine kinase* (*AsAHK*), which positively regulate the initiation of axillary meristem (Liu et al., 2020).

In conclusion, GAs positively regulate runner development in strawberry, whereas they negatively control shoot branching in tomato. In these species, GAs determine axillary bud outgrowth without influencing axillary bud initiation. Unlike the functions in strawberry and tomato, GAs promote axillary meristem initiation in garlic bulb. In addition, GAs act as pleiotropic regulators in two developmental stages of axillary meristem in many other horticultural species. *TEOSINTE BRANCHED1/BRANCHED 1* (*TB1*/*BRC1*) is the key hub in repressing axillary bud development in different species (Zhang et al., 2020). GAs and FaHAN can reduce this gene expression, leading to runner outgrowth in strawberry (Liang et al., 2022), while GAs possibly stimulate this gene expression *via* the cytokinin (CK) signal pathway, increasing shoot branching number in tomato (Xia et al., 2021). These results indicate that *BRC1* may be regulated differently in strawberry and tomato, and that the functions of GAs are also different in strawberry and tomato. Lateral Suppressor/LATERAL SUPPRESSOR/MONOCULM 1 (Ls/LAS/MOC1) determine axillary bud initiation (Feng et al., 2021). We predict that GAs maybe directly or indirectly influence the ortholog *Ls*/*LAS*/*MOC1* expression level in garlic bulb, and thus axillary bud initiation.

### Compound leaf

Leaf is the main plant photosynthetic organ (Du et al., 2018). There are three steps in leaf development: leaf primordium initiation, differentiation, and morphogenesis (Bar and Ori, 2015). Tomato has been used as a model species to study compound leaf development for decades and some progress has been achieved. Hormones are key factors regulating compound leaf development, and numerous studies have shown that GAs promote leaf differentiation, decreasing leaf complexity in tomato (Jasinski et al., 2008; Israeli et al., 2021; Su et al., 2022). For example, application of exogenous GAs or mutation of *PROCERA*/*DELLA* reduces the number of leaflets and makes leaf margins smoother and petioles longer (Jasinski et al., 2008; Fleishon et al., 2011; Yanai et al., 2011). However, CKs promote leaf morphogenesis and maintenance of organogenic activity, increasing leaflet number in tomato (Fleishon et al., 2011; Shwartz et al., 2016; Israeli et al., 2021). Overexpression of *ISOPENTENYL TRANSFERASE 7* (*IPT7*; a CK synthetic gene) increases leaf complexity (Fleishon et al., 2011). Compound leaf development is a complex process, so it is interesting to see how these two hormones maintain balance during compound leaf development.

**Figure 2:**
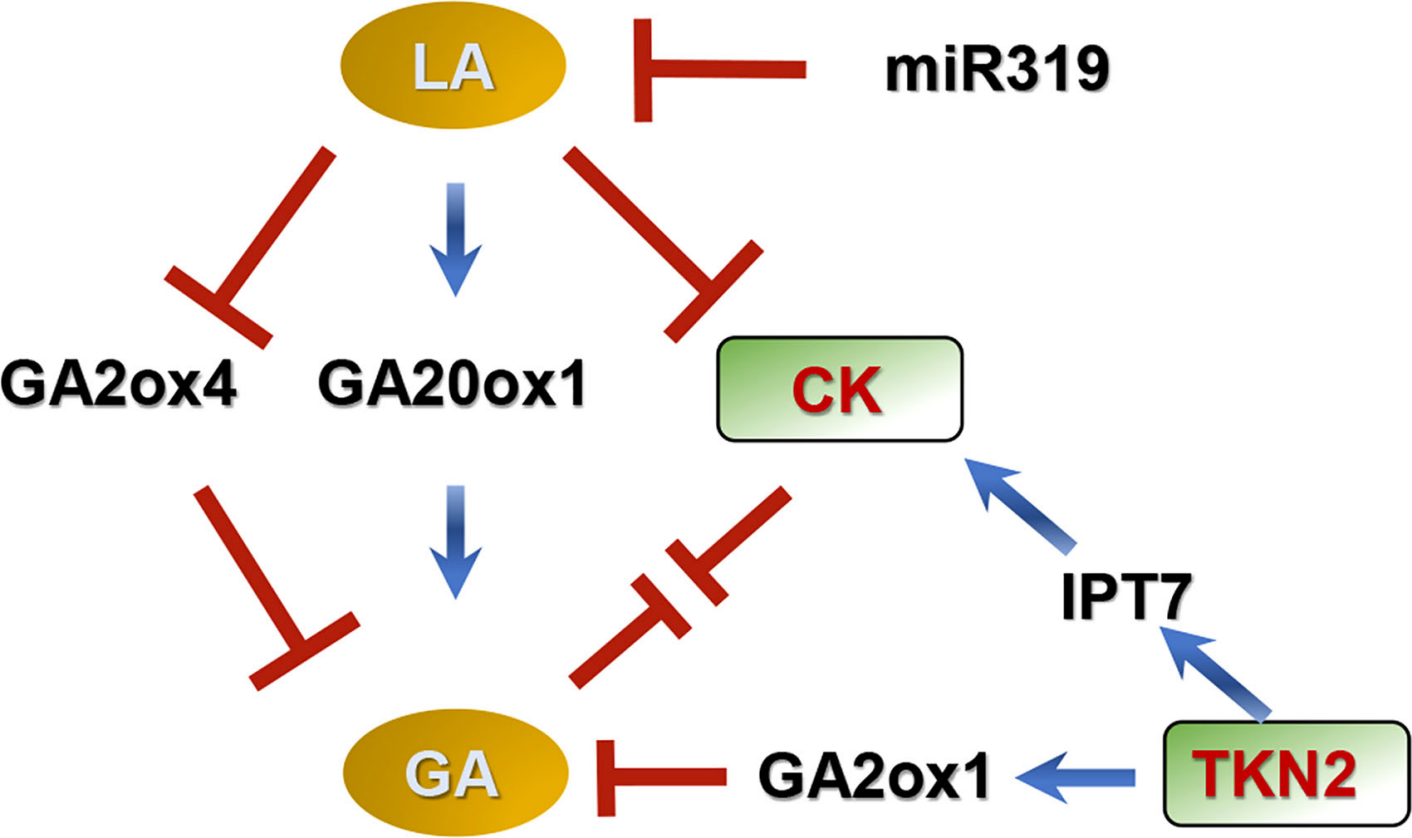
Model for compound leaf development regulated by GAs in tomato (Jasinski et al., 2005; Bolduc and Hake, 2009; Israeli et al., 2021). In this model, GAs promote differentiation and delay morphogenesis, decreasing leaf complexity, while CKs promote morphogenesis and delay differentiation, increasing leaflet number in tomato. GAs interact antagonistically with CKs during early leaflet origination. LANCEOLATE (LA) and TKN2 (a Class I KNOTTED1-LIKE HOMEOBOX protein) coordinate the balance between GAs and CKs during leaf development. *LA* is negatively regulated by MicroRNA319 (miR319). LA increases GA concentration by up-regulating *GA20ox1* and down-regulating *GA2ox4*, meanwhile this protein decreases CK concentration. TKN2 increases CK concentration by stimulating expression of *ISOPENTENYL TRANSFERASE 7* (a CK biosynthetic gene), and reduces GA concentration by motivating *GA2ox1* (a GA deactivated gene) expression. ↓ represents positive regulation. ⊥ represents negative regulation.

*Class I KNOX* (*KNOXI*) helps to maintain meristem activity, and delays leaf differentiation in many species (Hake et al., 2004; Cruz et al., 2020; He et al., 2020). Mutation of *TKN2* (a *KNOXI* gene) decreases leaf complexity in tomato, and the balance between GAs and CKs (organogenesis and differentiation) in leaflet formation is elaborately regulated by *TKN2* (**Figure 2**). In this model, *KNOXI* changes endogenous GA concentration by down-regulating *GA20ox1* and up-regulating *GA2ox* (Bolduc and Hake, 2009; Fleishon et al., 2011; Israeli et al., 2021). *KNOXI* also activates *IPT7*, leading to accumulation of CKs (Jasinski et al., 2005; Yanai et al., 2005; Fleishon et al., 2011; Israeli et al., 2021).

The MicroRNA319 (miR319)-LANCEOLATE (LA; a CIN-TCP TF) cascade also controls compound leaf development through coordinating GAs and CKs (**Figure 2**), in which miR319 promotes morphogenesis and LA promotes differentiation (Ori et al., 2007; Jasinski et al., 2008; Yanai et al., 2011; Challa et al., 2019; Israeli et al., 2021). *LA* is negatively controlled by miR319 during the early stages of leaf development, and overexpression of *miR319* causes an indeterminate growth phenotype of compound leaf (Ori et al., 2007; Jasinski et al., 2008; Yanai et al., 2011; Challa et al., 2019). The expression levels of *GA20ox1* and *GA2ox4* are respectively increased and decreased in the semi-dominant mutant *La*, indicating that *LA* increases GA levels (Yanai et al., 2011; Israeli et al., 2021). In addition, *LA* limits CK activity based on genetic experiments (Israeli et al., 2021).

In conclusion, early leaflet origination is a complex and dynamically balanced process. GAs interact antagonistically with CKs during this process, and promote the differentiation stage and shorten the morphogenetic stage, decreasing leaflet number. In contrast, CKs prolong morphogenesis and delay differentiation, increasing leaflet number. Multiple TFs such as TKN2 and LA delicately and fully alter concentrations of these hormones, changing the ratio of differentiation/morphogenetic stages and eventually influencing leaflet formation (**Figure 2**). The current model is a simple framework derived from limited existing data. Other TFs and hormones may also function in leaf development. A good example is CLAUSA (CLAU; a MYB TF), which works as LA in a mostly parallel pathway (Israeli et al., 2021). Overexpression of *CLAU* can rescue *LA* deficiency and vice versa. CLAU also medicate CK-GA balance in compound leaf development (Israeli et al., 2021). Auxins and brassinosteroids (BRs) can also coordinate with GAs, regulating compound leaf development in tomato. SlBES1.8, a key regulator of BR signaling, directly represses *SlGA2ox2*, *SlGA2ox6* and *SlGID1b-1*, influencing leaf morphogenesis (Su et al., 2022). Moreover, the specific molecular mechanism underlying compound leaf development may be different among different species (Bar and Ori, 2015). In pea, GAs prolong the morphogenetic stage, promoting leaf formation, which is different from the situation in tomato (Goliber et al., 1999). Current related studies have mostly focused on tomato; thus, it is important to study compound leaf development using other horticultural plants.

## Reproductive growth

### Flowering time

Transformation from vegetative to reproductive growth, which is one of the most important events in the plant life cycle, is a complex process regulated by many internal and external factors. The molecular mechanism underlying flowering has been studied extensively for decades, and there are five main pathways controlling floral induction in *Arabidopsis* (Fornara et al., 2010; Jin and Ahn, 2021). Among these five pathways, GAs accelerate floral induction *via* up-regulating *SOC1* in *Arabidopsis*, which is the so-called GA pathway (Mutasa-Gottgens and Hedden, 2009). This hormone is also involved in floral transition in horticultural plants. The difference in flowering time directly influences yield and quality of horticultural species (Randoux et al., 2012; Ghosh and Halder, 2018). Hence, it is important to explore the mechanism underlying how GAs regulate flowering transition of horticultural plants.

In tomato, DELLA coordinates microRNA156 (miR156)-SQUAMOSA PROMOTER BINDING–LIKE (SPL/SBP) cascade and miR319-LA cascade in determining flowering time through changing *SINGLE FLOWER TRUSS* (*SFT*) expression level in leaves and *APETALA1* (*AP1*) expression level in shoot meristem (Silva et al., 2019). Silva et al. (2019) showed that DELLA promotes flowering in tomato. In other words, GAs delay floral induction *via* reducing DELLA accumulation. Application of bioactive GAs decreases the expression levels of *AP1* and *FT* (known as a florigen gene) in *Citrus reticulata* Blanco × *Citrus temple* Hort. Ex Y. Tanaka (citrus) (Goldberg-Moeller et al., 2013), delaying flowering time. Once treated with GAs, *RoTERMINAL FLOWER1* (*RoTFL1*) transcripts are accumulated in once-flowering rose, down-regulating expression of *RoFT*, *RoSOC1*, and *RoAP1*, and inhibiting floral induction (Randoux et al., 2012). GAs also stimulate expression of *MdTFL1* in apple (Zhang et al., 2019). In contrast, the expression levels of *RsFT* and *RsSOC1-1* are up-regulated after GA treatment in early-flowering (NH-JS2) *Raphanus sativus* (radish; Jung et al., 2020). Recently, a GA-DELLA (SLR1)-VvmiR159c-*VvGIBBERELLIN MYB GENE* (*GAMYB*) cascade was reported to modulate grape floral development (Wang et al., 2018b). This result indicates that GAs also have a negative role in floral development in grape. In *Brassica campestris* L. ssp. *chinensis* var. *utilis* Tsen (flowering Chinese cabbage), GA treatment accelerates flowering and uniconazole (GA biosynthetic inhibitor) inhibits floral induction (Song et al., 2019).

Overall, GAs work as a negative factor in flowering transformation for tomato, rose, apple, and citrus, whereas they are a positive regulator in flowering Chinese cabbage, radish, and grape. GAs possibly influence, directly or indirectly, the expression of some core downstream floral integrator genes such as *AP1*, *SOC1*, and *FT*, determining the transformation of flowering (negative or positive). GAs thus have different roles in flowering transition among different horticultural species.

Some horticultural species, for example radish, are evolutionarily similar to *Arabidopsis* in effects of GAs on flowering time, while others such as tomato have evolved different or specific characteristics in regulation of flowering time. Because floral transition is controlled by a precise regulatory network, the difference in the effects of GAs on flowering time among different horticultural species may be associated with their different natural habitats and experimental conditions (Wang et al., 2021a). For example, GA treatment inhibits flowering in once-flowering rose in mid-March (short-day), but not in summer (long-day; Randoux et al., 2012). The seasonal differences in light period and temperature may be the reasons for the different role of GAs. These results provide clues for further study of the role of GAs on flowering time using different horticultural plant species. Tomato, citrus, and flowering Chinese cabbage are all short-day plants, while *Arabidopsis* is a long-day plant, which may be one of the reasons for the interspecific difference in the role of GAs. The mechanism underlying the effects of GAs on flowering time is largely unknown in various horticultural species, and thus more studies on flowering regulatory networks are needed, especially in economically important species.

### Parthenocarpy

Early fruit development of horticultural plants is a complex process, which is regulated by multiple phytohormones (Hu et al., 2018; Tyagi et al., 2021; Zhou et al., 2021; He and Yamamuro, 2022; Sharif et al., 2022). This process can be divided into three consecutive stages: fruit initiation stage, cell division stage controlled by auxins, and cell expansion stage regulated by GAs (Shinozaki et al., 2015). After fertilization, the developing seeds induce accumulation of GAs and auxins. Then, ovary or other tissues will receive GA and auxin signals from the immature seeds and activate the fruit initiation process (Hu et al., 2018).

Fruit formation without fertilization is also a common phenomenon, namely parthenocarpy. Parthenocarpy is an ideal agronomic trait for many horticultural species, and is popular with consumers. Recent studies show that numerous hormones are involved in parthenocarpic fruit formation (Martínez-Bello et al., 2015; Shinozaki et al., 2015; Hu et al., 2018; Liu et al., 2018; Zhou et al., 2021). A mass of cell cycle and cell expansion genes are continuously expressed in the ovary walls after treatment with phytohormones (Bermejo et al., 2016; Mesejo et al., 2016; Liu et al., 2018). Application of auxins or GAs can induce parthenocarpic fruit in tomato, cucumber, strawberry, and grape (Kang et al., 2013; Lu et al., 2016; Hu et al., 2018; Qian et al., 2018). These results demonstrate that auxins and GAs positively control parthenocarpy.

As parthenocarpic fruit growth is complicated, it is important to know how auxins coordinate with GAs during this process. AUX/IAA (IAA) proteins interact with AUXIN RESPONSE FACTOR (ARF) proteins to repress auxin signaling. In tomato, concentrations of bioactive GAs in parthenocarpic fruits increase in *iaa9* mutants (Serrani et al., 2008; Mignolli et al., 2015). Moreover, the numbers of transcripts of *SlGA20ox1* and *SlGA3ox1*, two GA synthesis genes, are increased in *SlARF7 RNAi* transgenic plants (Hu et al., 2018). *SlARF7* can directly bind to the promotors of *SlGA20ox1* and *SlGA3ox1*, and negatively regulate expression of these genes. PROCERA (DELLA) can also positively control expression of these genes (Hu et al., 2018). These results indicate that the interaction among PROCERA, SlARF7, and SlIAA9 coordinates the functions of auxins and GAs during parthenocarpic fruit growth. In strawberry, application of auxins also stimulates expression of *FveGA20ox* and *FveGA3ox* during growth of the parthenocarpic fruit formed from receptacle (Liao et al., 2018). Based on these results, we can conclude that auxins lead to GA accumulation by changing expression of GA biosynthesis genes in early stages of fruit development (Serrani et al., 2008; Mignolli et al., 2015; Hu et al., 2018; Zhou et al., 2021).

Although both auxins and GAs can stimulate parthenocarpic fruit formation, there are still some differences in the development process of the parthenocarpic fruits. For example, compared with normal fruits of strawberry the parthenocarpic fruits induced by auxins and GAs are rounder and longer, respectively (Liao et al., 2018). Auxins and GAs may regulate different genes related to cell division and expansion during parthenocarpic fruit development, leading to different fruit shapes (Liao et al., 2018; Chen et al., 2020).

Apart from auxins and GAs, CKs and ethylenes (ETHs) have also been reported to be involved in parthenocarpic fruit formation (Fos et al., 2000; Pascual et al., 2009; Shinozaki et al., 2015). CKs increase auxin concentrations, leading to parthenocarpic fruit in *Pyrus* spp. (pear) (Cong et al., 2020), while ETH decreases bioactive GA concentrations, inhibiting parthenocarpic fruit formation in tomato (Fos et al., 2000; Pascual et al., 2009; Shinozaki et al., 2015). However, a clear and systematic framework underlying the effects of various hormones on parthenocarpic fruit growth is still lacking. A recent review concluded that auxins may have a core role during this process (Sharif et al., 2022), but more evidence is needed to support this viewpoint in horticultural plants.

## A view to the future

Although some significant progress has been achieved regarding the roles of GAs in horticultural species, there are still many problems to be studied. Efficient genetic transformation and plant regeneration systems are still lacking for most horticultural species, limiting studies on the molecular mechanisms underlying the effects of GAs on growth and development in horticultural plants. It is worth constructing systems in multiple horticultural species in the future, which will certainly promote progress in relevant studies.

There are many GA synthetic or signaling mutants in horticultural plants, which is helpful not only to explore the roles of GAs, but also alter plant stature or other economic traits. However, unfavorable traits such as male sterility, poor germination, or low nitrogen-use efficiency are also found in some mutants (Zhang et al., 2014; Li et al., 2018a; He et al., 2019b), reducing their commercial value. There are two strategies for breeders to solve this challenge for horticultural species. First, *cis*-regulatory or coding sequences of a target GA synthetic or signaling gene can be edited using the CRISPR-Cas9 technique (Park et al., 2014; Wang et al., 2021b), and a series of alleles from weak to strong effects could be obtained (Zhang et al., 2020). The mild allele in plant height and other traits may be useful in altering agronomic traits of horticultural plants. Second, promoters with development stage- or tissue-specificity are urgently needed. A target GA synthetic or signaling gene can be driven by a stem-specific promoter, which may improve stem-related traits without effects on stamen or lateral roots development (Gupta et al., 2020). That is probably regarded as a prerequisite for the precision technique of manipulating traits of horticultural plants. In addition, the results obtained from laboratories should be tested in greenhouses or even in crop fields, accelerating use in practice.

## Author contributions

XZ and YS wrote the original manuscript. BZ provided help and advice. YF corrected the manuscript. All authors read and approved the submitted version.

## Funding

This study was supported by the National Natural Science Foundation of China (31971557 and 32171666) and the Scientific Research Project of Shenyang Agricultural University (880421034).

## Conflict of interest

The authors declare that the research was conducted in the absence of any commercial or financial relationships that could be construed as a potential conflict of interest.

## Publisher’s note

All claims expressed in this article are solely those of the authors and do not necessarily represent those of their affiliated organizations, or those of the publisher, the editors and the reviewers. Any product that may be evaluated in this article, or claim that may be made by its manufacturer, is not guaranteed or endorsed by the publisher.
